# Epidemiology of comorbidities and their association with asthma control

**DOI:** 10.1186/s13223-021-00598-3

**Published:** 2021-09-22

**Authors:** Gábor Tomisa, Alpár Horváth, Balázs Sánta, András Keglevich, Lilla Tamási

**Affiliations:** 1 Chiesi Hungary Ltd, Budapest, 1138 Hungary; 2 Department of Pulmonology, Semmelweis University, Tömő u. 25-29, Budapest, 1083 Hungary

**Keywords:** Asthma, Comorbidities, Age, Gender, BMI, Asthma control

## Abstract

**Background:**

The prevalence of comorbidities and their relation to asthma control and treatment is a topic of increasing interest, however comprehensive studies are scarce. We aimed to determine the prevalence of the most common comorbidities in asthma in relation to patient characteristics (age, gender and body mass index [BMI]) and their association with asthma control in a large, specialist-managed representative patient population.

**Methods:**

A secondary, exploratory analysis of the Asthma Reality (ARL), across-sectional, non-interventional real-life study was conducted. Basic patient characteristics, the prevalence of comorbidities and data on asthma control and risk factors had been collected and their interactions examined. Descriptive statistics and binomial regression were used to assess the distribution of the prevalence of comorbidities and propensity matching was applied to assess their effect on asthma control.

**Results:**

Overall, 12,743 patients were enrolled in our study in 187 treatment centres covering all regions of Hungary. Most comorbidities showed significantly different distribution for all basic patient characteristics. Gender, age group, smoking status, BMI and the duration of asthma had a significant impact on asthma control. The frequency of uncontrolled asthma was higher in females (37.1%), in the age group of 46–65 years (39.6%), in severely obese patients (43.2%), in patients who had been diagnosed with asthma for more than 20 years (40.4%), and in active heavy smokers (55%), compared with respective groups in the same category. Based on the binomial regression with propensity score matching, concomitant chronic obstructive pulmonary disease (COPD) (odds ratio [OR] = 2.06, 95% confidence interval [CI] 1.80–2.36), ischaemic heart disease (OR = 1.86, 95% CI 1.64–2.10) and cerebrovascular events (OR = 1.85, 95% CI 1.47–2.32) had the strongest negative effect on asthma control, with the presence of all of these conditions increasing the risk of uncontrolled asthma.

**Conclusions:**

This evaluation of comorbidity data of more than 12,000, adult asthmatic patients has provided a clearer picture of diseases that can frequently co-exist with asthma, and their influence on asthma control, assessed by the prevalence of symptoms. Our study suggests that most asthmatic patients have at least one comorbidity, and the presence of comorbidities may have a high impact on asthma control measures.

## Introduction

Asthma is a chronic inflammation of the airways characterized by reversible airflow obstruction and hyperreactivity of airways, which causes bronchospasms triggered by various endogenous and exogenous factors, allergens, or irritants [1–3]. Asthma is affecting more than 300 million people worldwide; this number could increase to 400 million by 2025 [4]. In Hungary, there were 316,098 adult asthmatic patients nationwide (not counting the paediatric population), which translates to an approximate prevalence of 3% [4]. This makes asthma one of the most common obstructive pulmonary diseases, with an even higher prevalence than COPD. However, in COPD, the prevalence and relevance of these comorbidities is well known, in asthma comorbidities are less defined and examined and may receive less attention during treatment. This gap in our knowledge is represented even in the current edition of Global Initiative for Asthma (GINA) treatment protocol which does not provide guidance on treatment modification in the context of the most common asthma comorbidities [1]. Although GINA mentions coexisting diseases, it does not elaborate on their exact prevalence, distribution, and role in determining asthma control and influencing patient management.

Since asthma cannot currently be cured (only treated), the emphasis in the treatment is on achieving asthma symptom control (symptoms typically include coughing, wheezing and shortness of breath) and preventing future asthma attacks. The definition of asthma severity is based on medication required to keep a patient in a well-controlled state, with more complex combination therapy or higher medication need indicating a more severe disease state.

We previously reported in a large, real-world evidence study (Asthma Reality [ARL]), risk factors for poor asthma outcomes and poor adherence, have a considerable influence on the control level of asthmatic patients [5]. As part of the ARL study, data on comorbidities had been collected. Due to the increasing interest in a more complex approach to treatment, we found that a database of this size (more than 12 thousand patients) is worth examining, especially because there had been no representative studies conducted in Hungary about the prevalence and possible influence of comorbidities on asthma control.

In this secondary, exploratory study, our goal was to determine the prevalence of the most common comorbidities in asthma in relation to patient characteristics—age, gender, and body mass index (BMI)—and their possible relation with asthma control in a large, specialist-managed representative patient population.

## Methods

Details of the ARL study [5] were previously published, but are briefly described here.

### Trial design

This was a non-interventional, cross-sectional real-life study. Inclusion of the patients and data recording was performed on a single occasion. For detailed data collection purposes, separate doctor and patient questionnaires were developed. To eliminate seasonal effects, patient recruitment was executed throughout an entire year (from May 2015 to May 2016). To obtain a non-biased patient enrolment, every health institution included a maximum of 15 patients on five predetermined consecutive workdays per month. Enrolment was conducted randomly with the inclusion of consecutive asthmatic patients after they provided informed consent. Dispensaries, outpatient clinics specialising in pulmonology and outpatient departments of hospitals in all regions of Hungary participated in the study. Table 1 contains the inclusion and exclusion criteria of the study. The design and the implementation of the study were conducted according to good clinical practice (GCP) guidelines and the Declaration of Helsinki.

**Table 1:** Inclusion and exclusion criteria [5]

Inclusion criteria
Adult asthmatic patients (> 18 years)
Asthma diagnosis for > 6 months
Maintenance therapy unchanged in the last month
Outpatient
No hospitalisation in the last month
No prominent, untreated chronic disease

Exclusion criteria
Lack of patient consent
Inability to complete patient-related questionnaires
Permanent need for maintenance systemic corticosteroid treatment
Acute exacerbations at time of inclusion in the study
Active tuberculosis
Malignant disease in a palliative treatment phase

### Recorded data

A comprehensive data collection form was used to record patient demographic characteristics, major medical history, smoking habits, comorbidities, risk factors, current control state, medications, and all relevant physical assessments. Asthma control, treatment steps, and risk factor assessment were done according to GINA 2014 (see Table 2) [6]. BMI was calculated based on the patient’s measured height and weight at the time of examination. The evaluated comorbidities were the following: seasonal and perennial rhinitis, gastroesophageal reflux disease (GERD), COPD, hypertension, atrial fibrillation, other arrhythmias, ischaemic heart disease (IHD), acute myocardial infarction (AMI), ‘other cardiac events’, cerebrovascular events, osteoporosis, diabetes mellitus (DM), impaired fasting glucose (IFG—which means an increase in fasting blood glucose level, with the glucose tolerance retained) [7], prostate hyperplasia, glaucoma and ‘other comorbidities’ (an umbrella term for all other comorbidities not specifically listed in the questionnaire). As cardiovascular diseases are one of the most prevalent diseases in the general population and are the leading cause of mortality in Hungary [8], we aimed to evaluate them in a more detailed manner. For that purpose, in addition to examining their prevalence individually, a combined comorbidity index was created (‘cardiovascular diseases’) encompassing the following diseases: hypertension, atrial fibrillation, other arrhythmias, IHD, AMI, and ‘other cardiac events’. Given that the main objective of this study was to evaluate the presence of comorbidities, a secondary check on recorded data was performed and minor mistakes in data recording were corrected (the number of prostate hyperplasia changed from 317 patients to 306). In addition, for ‘other arrhythmias’, we concluded that the diagnoses of 107 patients were not medically confirmed.

**Table 2:** [6] Definitions of controlled, un- and partially controlled asthma according to GINA 2014

	Controlled	Partially controlled	Uncontrolled
Daytime asthma symptoms more than twice a week? Any night waking due to asthma? Reliever needed for symptoms more than twice a week?* Any activity limitation due to asthma?	None of these	1–2 of these	3–4 of these

^*^The only difference compared to GINA 2021 is that the latter defines reliever need only as SABA reliever need, as-needed ICS-formoterol use is not to be considered as increased need for reliever therapy [1, 6]

### Objectives

Our main objective was to evaluate the prevalence of various comorbidities and their association with asthma control in an outpatient, specialist-managed asthmatic patient population. We also aimed to uncover the relationship between basic patient characteristics to comorbidities and to asthma control. For that purpose, data on gender, age, BMI, duration of asthma diagnosis, smoking history, history of comorbidities, and current asthma control were collected. Since our aim was to uncover more robust trends, controlled and partially controlled patients were grouped together as controlled. To distinguish the effect of comorbidities on asthma control from the aforementioned confounding factors, a multivariate approach was used.

### Statistical analysis

Data collection and database management were conducted by AdWare Research Ltd. (Balatonfüred, Hungary); the statistical analysis was conducted by Adatrendező Ltd. (Dunaharaszti, Hungary). In the data analysis phase, descriptive statistics, graphical outputs, Fisher’s exact test, analysis of variance (ANOVA), and the chi-square test of independence were used. Odds ratios (ORs) were provided with 95% confidence intervals (CIs). In the case of smoking history, patients were grouped according to smoking exposure (measured in pack-years) and smoking status (nonsmoker, active smoker, and past smoker), and the combination of these parameters. Propensity scores were created based on gender, age group, BMI, time since asthma diagnosis and smoking history. Propensity scores are commonly used for stratifying patients into groups with similar characteristics (based on the parameters incorporated in the propensity score). In observational studies, there is no opportunity to randomize patients. However, matching them into groups with similar propensity scores helps to eliminate the confounding effects of differences in baseline parameters. In our case comparing the asthma control of patients with or without a certain comorbidity, in the same propensity score group allows for the comparison of the effect of the comorbidity, free (or at least mostly free) from the confounding effect of baseline characteristics (BMI, age etc.) on asthma control. This method allows for the most accurate analysis of the relation of comorbidities to asthma control in an observational study.

For statistical analysis, we used the open-source Python 2.7.12 on a MAC operating system (Anaconda Inc., Austin, TX) and R for Windows 3.4.2 (R Core Team, https://www.R-project.org/).

## Results

### Patients

During the 1-year period of inclusion, an average of 69 patients were included per investigational site. This involved 187 centres, representing 35% of the pulmonologists currently working in outpatient medical clinics in Hungary [5].

We studied the prevalence of common noncommunicable diseases coexisting with asthma in the context of the aforementioned patient characteristics. The basic demographic characteristics are shown in Table 3 and details on comorbidities present are in Table 4A and B.

**Table 3:** Numbers and proportions of patients with controlled and uncontrolled asthma in each demographic parameter

		Controlled	Uncontrolled	p values*
Gender	Male (n = 4059)	2858 (70%)	1201 (30%)	0.0000
	Female (n = 8684)	5462 (63%)	3222 (37%)	
Age group	18–30 (n = 1261)	931 (74%)	330 (26%)	0.0000
	31–45 (n = 2466)	1812 (73%)	654 (27%)	
	46–65 (n = 5687)	3437 (60%)	2250 (40%)	
	> 65 (n = 3329)	2140 (64%)	1189 (36%)	
BMI	Underweight (n = 228)	145 (64%)	83 (36%)	0.0000
	Normal (n = 3475)	2419 (70%)	1056 (30%)	
	Overweight (n = 4465)	2967 (66%)	1498 (34%)	
	Obese (n = 3009)	1900 (63%)	1109 (37%)	
	Severly obese (n = 1566)	889 (57%)	677 (43%)	
Smoking amount	Non smoker (n = 8432)	5759 (68%)	2673 (32%)	0.0000
	< 10 pack-years (n = 2077)	1356 (65%)	721 (35%)	
	≥ 10 pack-years (n = 2234)	1205 (54%)	1029 (46%)	
Smoking status	Active smoker (n = 1669)	963 (58%)	706 (42%)	0.0000
	Ex-smoker (n = 2588)	1568 (61%)	1020 (39%)	
	Non smoker (n = 8486)	5789 (68%)	2697 (32%)	
Time since diagnosis	0–5 years (n = 4222)	2915 (69%)	1307 (31%)	0.0000
	6–10 years (n = 3174)	2047 (64%)	1127 (36%)	
	11–20 years (n = 3617)	2326 (64%)	1291 (36%)	
	> 20 years (n = 1702)	1014 (60%)	688 (40%)

**Table 4:** A and B Numbers and proportions of patients with controlled and uncontrolled asthma in each comorbidity

		Controlled	Uncontrolled
A			
Cardiovascular diseases	Not present (n = 7160)	5084 (71%)	2076 (29%)
	Present (n = 5583)	3236 (58%)	2347 (42%)
Seasonal rhinitis	Not present (n = 10,957)	7331 (67%)	3626 (33%)
	Present (n = 1786)	989 (55%)	797 (45%)
Perennial rhinitis	Not present (n = 4226)	2766 (65%)	1460 (35%)
	Present (n = 8517)	5554 (65%)	2963 (35%)
GERD	Not present (n = 10,180)	6847 (67%)	3333 (33%)
	Present (n = 2563)	1473 (57%)	1090 (43%)
COPD	Not present (n = 11,741)	7869 (67%)	3872 (33%)
	Present (n = 1002)	451 (45%)	551 (55%)
Ischaemic heart disease	Not present (n = 11,493)	7704 (67%)	3789 (33%)
	Present (n = 1250)	616 (49%)	634 (51%)
Hypertension	Not present (n = 7517)	5285 (70%)	2232 (30%)
	Present (n = 5226)	3035 (58%)	2191 (42%)
AMI	Not present (n = 12,533)	8211 (66%)	4322 (34%)
	Present (n = 210)	109 (52%)	101 (48%)
Atrial fibrillation	Not present (n = 12,583)	8233 (65%)	4350 (35%)
	Present (n = 160)	87 (54%)	73 (46%)
B			
Other arrhythmias	Not present (n = 12,119)	7999 (66%)	4120 (34%)
	Present (n = 624)	321 (51%)	303 (49%)
Other CV	Not present (n = 12,528)	8215 (66%)	4313 (34%)
	Present (n = 215)	105 (49%)	110 (51%)
Cerebrov. events	Not present (n = 12,432)	8171 (66%)	4261 (34%)
	Present (n = 311)	149 (48%)	162 (52%)
Osteoporosis	Not present (n = 11,662)	7715 (66%)	3947 (34%)
	Present (n = 1081)	605 (56%)	476 (44%)
Impaired fasting glucose	Not present (n = 11,685)	7765 (66%)	3920 (34%)
	Present (n = 1058)	555 (52%)	503 (48%)
Diabetes mellitus	Not present (n = 11,614)	7726 (67%)	3888 (33%)
	Present (n = 1129)	594 (53%)	535 (47%)
Prostate hyperplasia	Not present (n = 12,437)	8131 (65%)	4306 (35%)
	Present (n = 306)	189 (62%)	117 (38%)
Glaucoma	Not present (n = 12,516)	8196 (65%)	4320 (35%)
	Present (n = 227)	124 (55%)	103 (45%)
Other	Not present (n = 11,423)	7607 (67%)	3816 (33%)
	Present (n = 1320)	713 (54%)	607 (46%)

*AMI* acute myocardial infarction, *Cerebrov.* cerebrovascular events, *COPD* chronic obstructive pulmonary disease, *CV* cardiovascular, *GERD* gastroesophageal reflux disease

### Gender

**Fig. 1:**
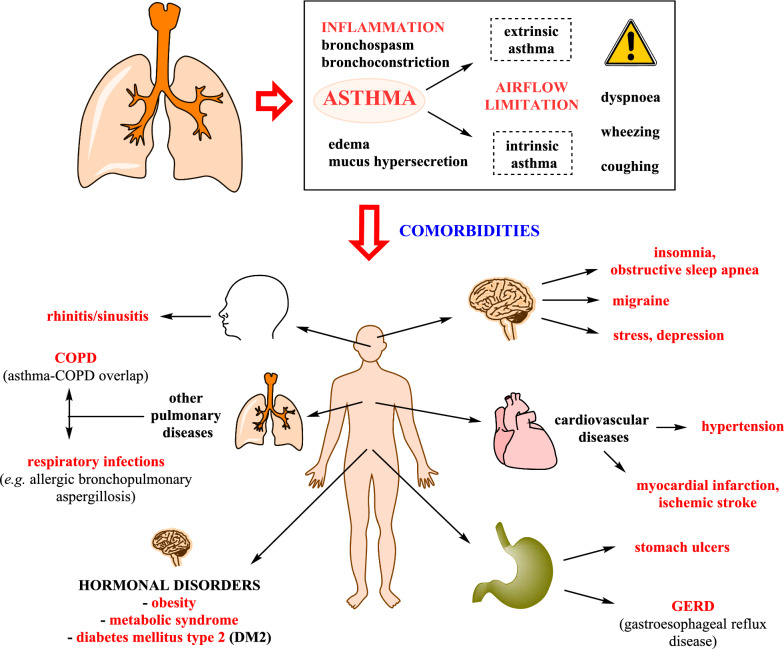
The inflammatory cells and mediators in the pathogenesis of asthma (above) [3, 35], and the most frequently reported comorbid conditions (below) [11–13]. COPD = chronic obstructive pulmonary disease

The majority of the patients in our study were female (68.1%), similar to the gender distribution of the general adult asthmatic patient population in Hungary [6]. There was a statistically significant difference in the mean age of female compared with male patients (55.2 vs 51.5 years; p < 0.0001). The following comorbidities were significantly more prevalent in female patients (percentage of female population vs percentage of male population; p value): cardiovascular diseases (46.7% vs 37.7%; p < 0.0001), GERD (22.2% vs 15.6%; p < 0.0001), hypertension (43.9% vs 34.9%; p < 0.0001), other arrhythmias (6.1% vs 5%; p = 0.0034), osteoporosis (11.5% vs 2.1%; p < 0.0001), IFG (8.7% vs 7.5%; p = 0.0210), glaucoma (2.1% vs. 1.2%; p = 0.0004) and ‘other comorbidities’ (11.9% vs 7.1%; p < 0.0001). The following diseases were found to be significantly more prevalent in male patients: concomitant COPD (9.3 vs 7.2%; p = 0.0001), AMI (2.3 vs 1.4%; p = 0.002) and atrial fibrillation (1.6 vs. 1.1%; p = 0.0208). Furthermore, the proportion of uncontrolled asthma was significantly higher in females (37.1% vs 29.6%; p < 0.0001) (Fig. 1).

### Age

**Fig. 2:**
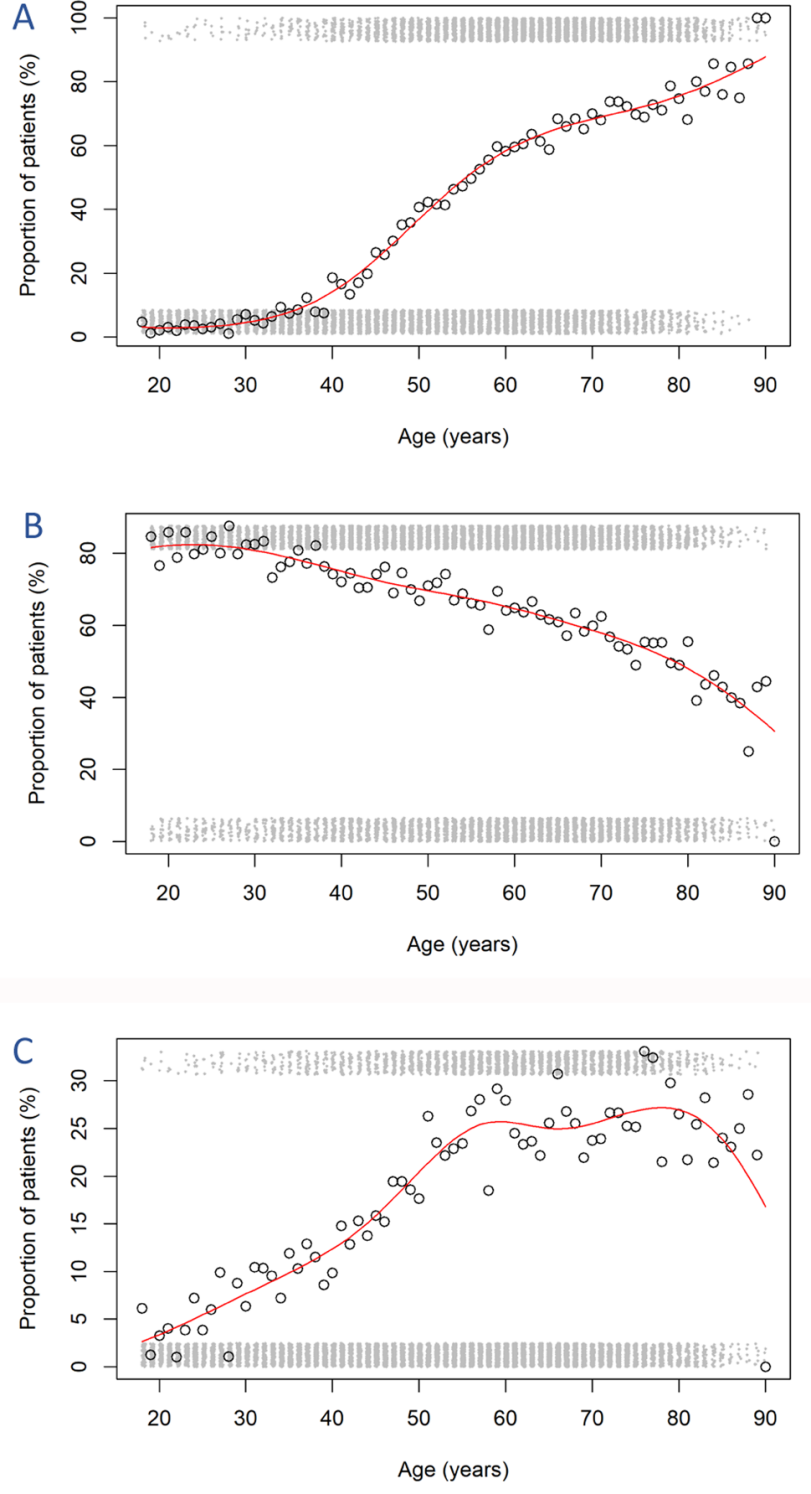
The distribution of patients with cardiovascular disease (age trend 1, **A**), seasonal rhinitis (age trend 2, **B**) and gastroesophageal reflux disease (GERD) (age trend 3, **C**)

The distribution of patients into groups according to age was the following: 18–30 years old (9.9%; age group 1), 31–45 years old (19.4%; age group 2), 46–65 years old (44.6%; age group 3) and > 65 years old (26.1%; age group 4). Older patients tended to have more comorbidities than younger ones; however certain diseases showed a different distribution according to age. To aid understanding, we grouped comorbidities into three categories: diseases with age-related increasing prevalence (trend 1), decreasing prevalence (trend 2), and diseases whose age distribution showed a different pattern (trend 3). Figure 2 shows an example of a comorbidity that follows each trend.

#### Trend 1

Cardiovascular diseases were present in 3.5% of all patients in group 1 while 70.4% of patients suffered from them in group 4, with a steep increase in prevalence starting at 50 years of age. A similar trend was observed for IHD (prevalence of 0.2% in group 1 and 20.4% in group 4), AMI (from 0.2 to 3.0%), atrial fibrillation (from 0.24 to 3.18%), other arrhythmias (from 0.6 to 8.4%), other cardiovascular events (from 0.4 to 3.2%), cerebrovascular events (from 0.1 to 4.7%) and hypertension (from 2.4 to 66.4%). Concomitant COPD showed an increase up to 12.6% in group 4 (vs 0.4% in group 1). Osteoporosis mostly occurred at the age of 40 and increased in prevalence, reaching 17.9% in group 4. Prostate hyperplasia (from 0.0% in group 1 to 6.8% in group 4), IFG (from 0.6 to 13.2%), DM (from 0.8 to 13.9%) and glaucoma (from 0.2 to 3.2%) also showed increased prevalence with age in asthmatic patients.

#### Trend 2

Contrary to the aforementioned diseases, the prevalence of perennial rhinitis (from 82.3% in group 1 to 55.5% in group 4) decreased with age.

#### Trend 3

The occurrence of GERD increased until the age of 55, when it reached 25% and stayed the same after, regardless of increasing age. ‘Other comorbidities’ showed a trend with a similar plateau starting from the age of 50 years. Furthermore, there was a statistically significant difference in the mean age of patients with controlled asthma (53.1 years, 95% CI 52.4–53.4) and uncontrolled asthma (55.9 years, 95% CI 55.4–56.3; p < 0.0001). The proportion of patients with uncontrolled asthma was highest in age group 3 (39.6%).

### BMI

Patients were categorised into five groups according to BMI values: underweight (BMI ≤ 18.5 kg/m^2^; 1.8%), normal (BMI 18.5–25 kg/m^2^; 27.3%), overweight (25–30 kg/m^2^; 35.0%), obese (30–35 kg/m^2^; 23.6%) and severely obese (> 35 kg/m^2^; 12.3%).

The average BMI was significantly higher in patients with the following comorbidities: cardiovascular disease, hypertension, IHD, AMI, atrial fibrillation, other arrhythmias, DM, IFG, GERD and other comorbidities (in all cases, p < 0.0001); glaucoma (p = 0.001); other cardiovascular events (p = 0.001); cerebrovascular disease (p = 0.004); and prostate hyperplasia (p = 0.049). On the other hand, the average BMI was significantly lower in patients with perennial (p < 0.0001) and seasonal (p = 0.019) rhinitis.

The prevalence of most evaluated comorbidities increased with BMI, with the largest increase observed in the rate of hypertension (present in 8.3% of underweight and 62.1% of severely obese patients). A similar BMI-related increase was seen in the prevalence of IHD, AMI, atrial fibrillation, other arrhythmias, other cardiovascular events, DM, IFG, and ‘other comorbidities’. There was a similarly increasing prevalence for GERD, with a plateau at a BMI of 30 kg/m^2^.

There was a reversed trend for perennial and seasonal rhinitis, where the prevalence decreased as the BMI increased. In the case of the much more prevalent perennial rhinitis, 71.9% of underweight and only 61.9% of severely obese patients were affected.

There was a trend for an increasing then decreasing prevalence for cerebrovascular events, prostate hyperplasia, and glaucoma.

The prevalence of concomitant COPD and osteoporosis were independent of BMI values.

Finally, there was a significant difference in the average BMI of the patients with controlled asthma (28.1 kg/m^2^, 95% CI 28.0–28.2) and uncontrolled asthma (29.1 kg/m^2^, 95% CI 29.0–29.3; p < 0.0001). The severely obese patients had the highest proportion of uncontrolled asthma (43.2%).

### Duration of asthma

33% of our patients had been diagnosed with asthma for 0–5 years, 25% for 6–10 years, 28% for 11–20 years, and 13% for more than 20 years.

Eight of the assessed comorbidities showed no correlation with the duration of asthma (GERD, IHD, concomitant COPD, cerebrovascular episodes, elevated blood sugar, DM, glaucoma, and other arrhythmias). By contrast, the prevalence of patients with perennial rhinitis, hypertension, other cardiac episodes, osteoporosis, prostate hyperplasia, and other comorbidities correlated with the duration of asthma. On the other hand, the prevalence of AMI and atrial fibrillation was lower in patients who had had their asthma diagnosis for a longer time.

There was a statistically significant difference in the duration of asthma of patients with controlled asthma (10.6 years, 95% CI: 10.5–10.8) and uncontrolled asthma (11.9 years, 95% CI: 11.6–12.2; p < 0.0001). The patients who had been diagnosed for more than 20 years had the highest proportion of uncontrolled asthma (40.4%).

### Smoking

Thirteen percent of the patients were active smokers, 20.3% were ex-smokers and 66% of them had never smoked.

The data on smoking history and exposure were analysed separately and combined. Overall, 66.2% (8,432) of the patients were nonsmokers, while 4.8% (616) smoked < 10 pack-years (‘moderate smoker’) and 8.3% (1053) smoked > 10 pack-years (‘heavy smoker’). Finally, 11.5% (1461) of the patients were ex-smokers, 11.5% (1461) with less than 10 pack-years of exposure and 9.3% (1,181) were ex- and heavy smokers.

There was a statistically significant difference in the average age of heavy versus moderate and of active smokers versus ex-smokers. Taking into account that the prevalence of comorbidities is affected by age, we incorporated this variable into our calculations when assessing the effect of smoking habits on the prevalence of comorbidities. Thus, we compared the prevalence of certain comorbidities between smokers, ex-smokers and nonsmokers, as well as between heavy and moderate smokers of the same age.

### Smoking status

There was a statistically significant overall difference between smokers, ex-smokers and nonsmokers in the prevalence of the following comorbidities: COPD, perennial rhinitis, AMI, IFG, DM, prostate hyperplasia, cardiovascular diseases (in all cases, p < 0.0001); other arrhythmias (p = 0.0003); other cardiovascular events (p = 0.001); other comorbidities (p = 0.001); IHD (p = 0.001); osteoporosis (p = 0.002); seasonal rhinitis (p = 0.003); and hypertension (p = 0.007). Based on pairwise comparisons, ex-smokers had the highest disease burden in nine of the 14 aforementioned comorbidities, with a statistically significantly higher prevalence compared with nonsmokers in hypertension, IHD, DM, cardiovascular diseases, other cardiovascular events and other arrhythmias, and a statistically significantly higher prevalence compared with nonsmokers and active smokers in IFG, prostate hyperplasia and AMI. For seasonal rhinitis, osteoporosis, other comorbidities and COPD, active smokers had the highest prevalence compared with ex-smokers and nonsmokers. It is interesting to note that perennial rhinitis was most prevalent in nonsmokers and least prevalent in active smokers (statistically significant difference between all groups).

### Smoking exposure

There was a statistically significant overall difference between heavy, moderate and nonsmokers in the prevalence of the following diseases: COPD, perennial rhinitis, AMI, IFG, DM, prostate hyperplasia, seasonal rhinitis (in all cases, p < 0.0001); other arrhythmias (p = 0.0004); cardiovascular diseases (p = 0.0005); IHD (p = 0.003); other cardiovascular events (p = 0.008); GERD (p = 0.027); cerebrovascular diseases (p = 0.033); and hypertension (p = 0.033). Based on the pairwise comparisons of the 15 aforementioned conditions, 14 were significantly more prevalent in the heavy smoker group, 12 were more prevalent compared with nonsmokers and two (GERD and cerebrovascular disease) were more prevalent compared with moderate smokers. The difference was statistically significant compared with nonsmokers and moderate smokers for IFG, DM, seasonal rhinitis, and other comorbidities. Moreover, the prevalence of COPD, AMI, other arrhythmias, and prostate hyperplasia was significantly higher in heavy smokers compared with moderate smokers, and in moderate smokers compared with nonsmokers, suggesting a dose-dependent effect. Interestingly, perennial rhinitis showed a reverse trend, with a significantly higher prevalence in nonsmokers compared with both moderate and heavy smokers.

### Combined analysis

To assess clearly the exact effect of smoking status and exposure on the prevalence of comorbidities in asthma, a combined analysis of the two parameters was performed. For this analysis, patients were assigned to one of the following five groups: NS (nonsmoker; 66.2%), ES1 (ex-smoker with < 10 pack-years of exposure; 11.5%), AS1 (active smokers, < 10 pack-years; 4.8%), ES2 (ex-smoker, > 10 pack-years; 9.3%) and AS2 (active smoker, > 10 pack-years; 8.3%).

There was a statistically significant overall difference in the prevalence of the following diseases among the aforementioned groups: other comorbidities, AMI, seasonal rhinitis, perennial rhinitis, concomitant COPD, IFG, DM, prostate hyperplasia (in all cases, p < 0.0001); cardiovascular disease (p = 0.0002); osteoporosis (p = 0.002); other arrhythmias (p = 0.003); other cardiovascular events (p = 0.004); GERD (p = 0.007); hypertension (p = 0.008); IHD (p = 0.014); and cerebrovascular diseases (p = 0.041).

Based on the pairwise comparisons, the group most affected by comorbidities was ES2. The prevalence of 11 of the aforementioned comorbidities was significantly higher in this group compared with all other groups (IFG, DM, and other comorbidities), or to nonsmokers (AMI, prostate hyperplasia, cardiovascular diseases, hypertension, IHD, other arrhythmias, and other cardiovascular events). COPD, AMI, other comorbidities, and seasonal rhinitis had a higher prevalence in the active smoker groups, especially in AS2 (except for AMI, which was most prevalent in AS1). Interestingly, the prevalence of GERD was lowest in AS1; this was the only group with a statistically significant difference in prevalence compared with any other groups. While the prevalence of COPD significantly increased with more exposure and active smoking (highest in AS2), similarly to the previous analysis, perennial rhinitis showed a reverse pattern, with nonsmokers having the highest prevalence compared with ES1, ES2, AS1, and AS2. Indeed, AS2 showed the lowest prevalence of all groups.

There was no significant difference in the prevalence of atrial fibrillation and glaucoma among nonsmokers and smokers of any definition. Furthermore, the risk of having uncontrolled asthma was highest in active and heavy smokers and lowest in nonsmokers, with a statistically significant difference in prevalence also in the combined analysis. AS2 (numerically the highest prevalence) and ES2 patients had the worst asthma control compared with all other groups.

### The effect of comorbidities on asthma control measures

The main goal of our analysis was to assess the possible effects of comorbidities on asthma control and treatment outcomes. We used propensity scores to mitigate the confounding effect of basic patient characteristics for the prevalence of the given comorbidity as well as the treatment outcomes. With this method, patients were classified into five groups within which they only differed in the presence or absence of the evaluated comorbidity (as if they were randomised). The effect of the presence of the given comorbidity and each component of the propensity score on asthma control was evaluated and ORs were calculated.

**Fig. 3:**
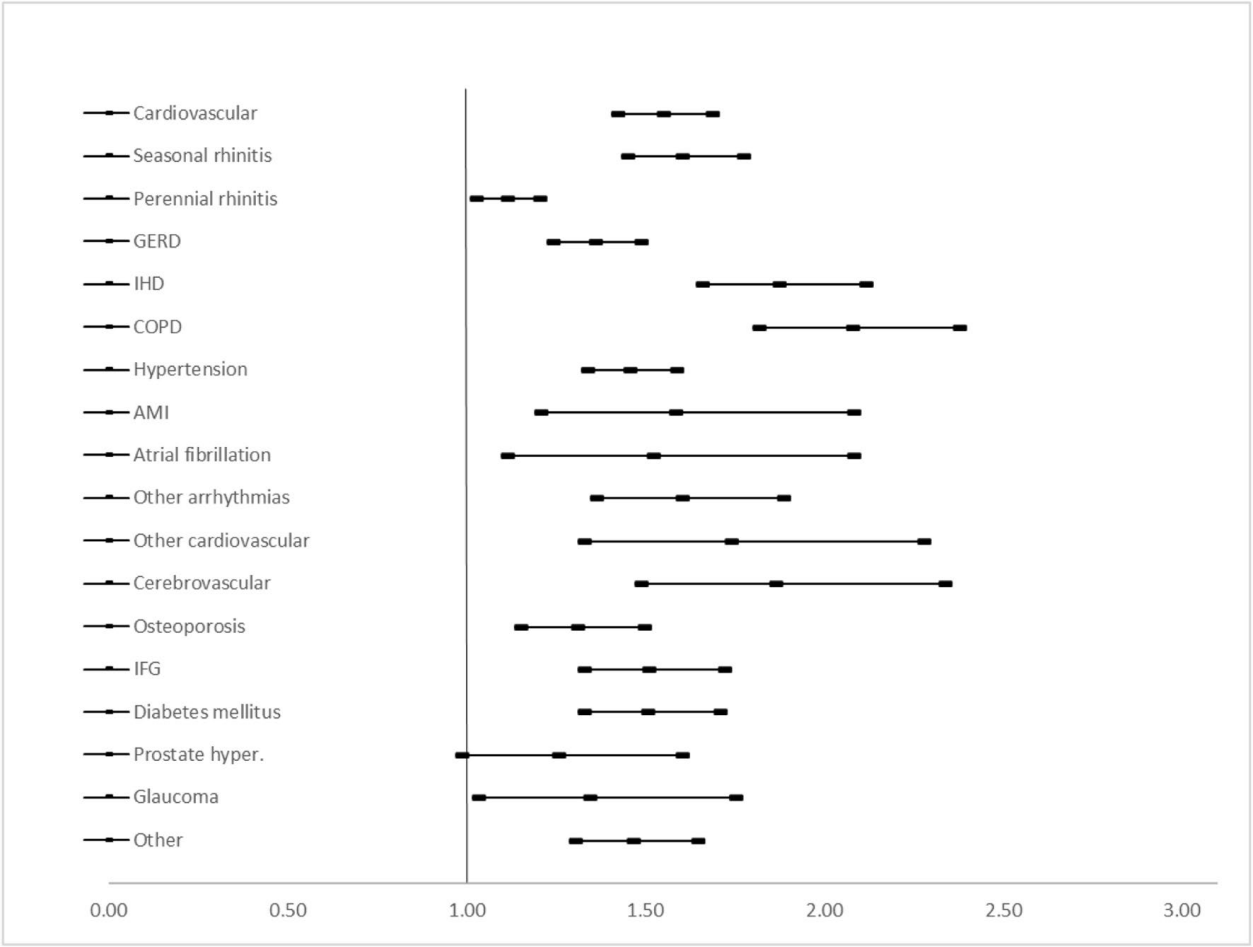
Risk of having uncontrolled asthma if the given comorbidity is present

**Fig. 4:**
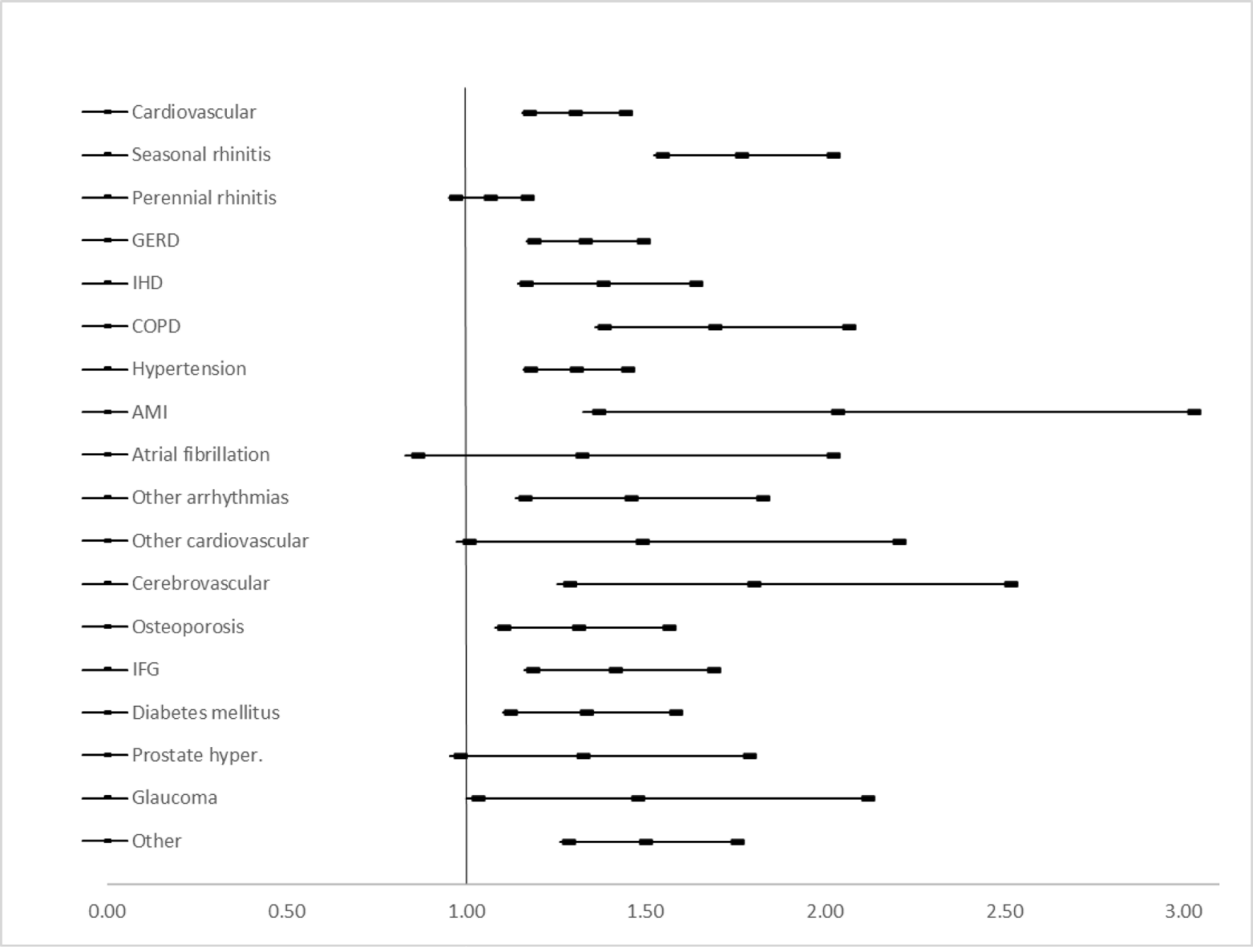
Risk of having partially controlled asthma if the given comorbidity is present

**Fig. 5:**
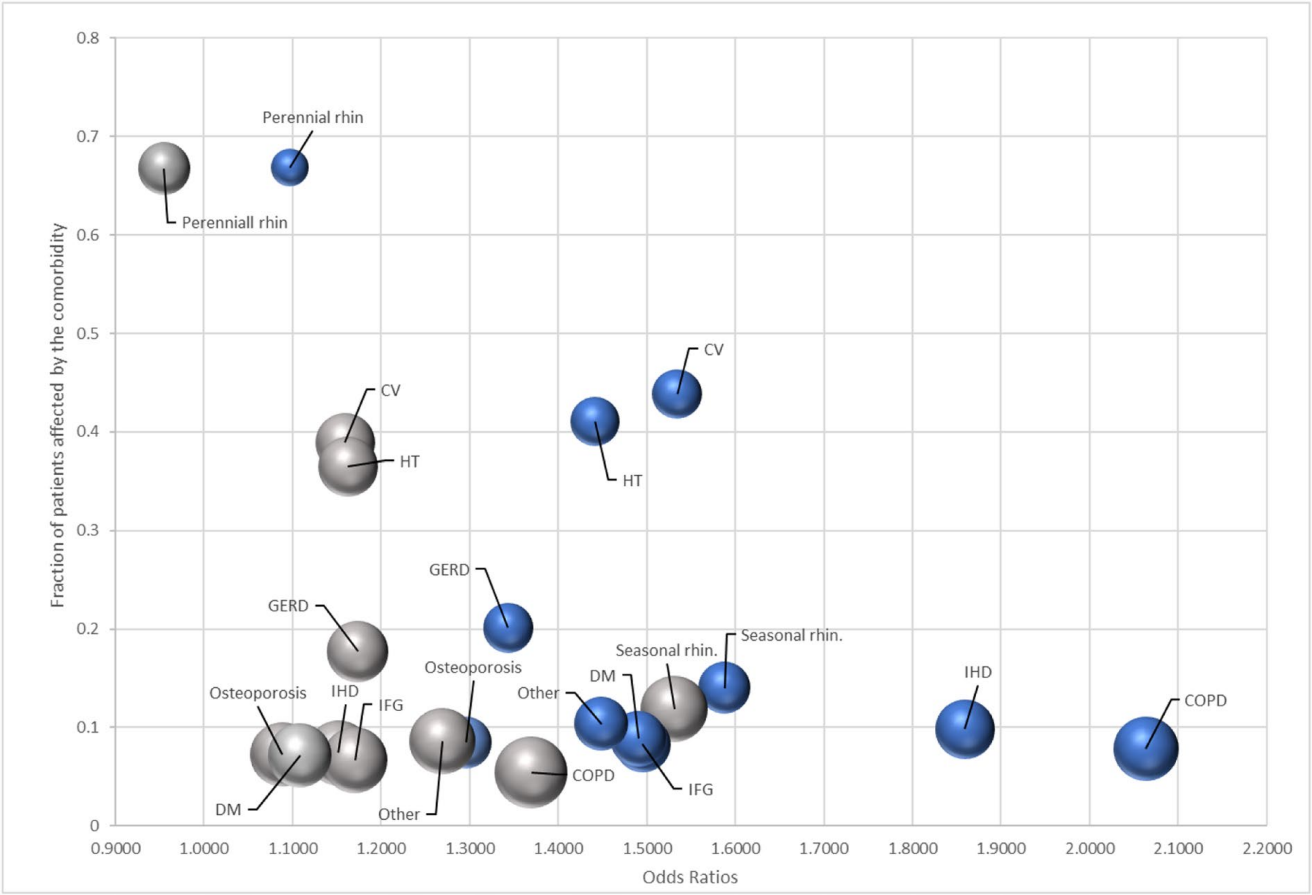
The frequency of specific comorbidities (prevalence of at least 10%) and the odds ratios of their relationship with uncontrolled (blue) and partially controlled (grey) asthma. The size of the bubbles represents the ratio of uncontrolled asthma (of all patients who suffer from that specific comorbidity). COPD = chronic obstructive pulmonary disease; CV = cardiovascular; DM = diabetes mellitus; GERD = gastroesophageal reflux disease; HT = hypertension; IFG = impaired fasting glucose; IHD = ischaemic heart disease; rhin. = rhinitis

Asthma control was evaluated as controlled (comprising well and partially controlled, n = 8320) versus uncontrolled (n = 4423), and within the whole controlled group as well controlled (n = 4588) versus partially controlled (n = 3732) asthmatic patients. For the first comparison (Fig. 3), all comorbidities had a statistically significant effect on asthma control levels, except for prostate hyperplasia (OR = 1.24, 95% CI 0.97–1.59). Concomitant COPD (OR = 2.06, 95% CI 1.80–2.36), IHD (OR = 1.86, 95% CI 1.64–2.10) and cerebrovascular events (OR = 1.85, 95% CI 1.47–2.32) had the strongest effect on treatment outcome. Comparing well versus partially controlled asthma (Fig. 4), prostate hyperplasia (OR = 1.31, 95% CI 0.97–1.77), atrial fibrillation (OR = 1.31, 95% CI 0.85–2.01), perennial rhinitis (OR = 1.05, 95% CI: 0.96–1.16) and other cardiovascular events (OR = 1.48, 95% CI 0.99–2.19) had a significant effect on asthma control level. AMI (OR = 2.02, 95% CI 1.35–3.01), cerebrovascular events (OR = 1.79, 95% CI 1.27–2.50) and seasonal rhinitis (OR = 1.75, 95% CI 1.53–2.01) had the strongest effect on the control level in this comparison (Fig. 5).

## Discussion

The presence of asthma might increase the risk of certain comorbidities [9, 10] that may worsen asthma symptoms and provoke acute flare-ups, especially if they are not treated appropriately [10–13]. Thus, the GINA guidelines recommend assessing comorbidities before treatment modification if asthma control worsens [1]. However, neither the GINA nor other guidelines provide detailed recommendations on the assessment of comorbidities or provide information on only a subpopulation of all asthmatic patients [14–16]. In our study, we evaluated the prevalence of 18 different comorbidities, their distribution in the population and their effect on asthma control levels. Our aim was to provide a comprehensive data set of the most prevalent diseases that can affect asthmatic patients and, simultaneously, to compare their effect on asthma treatment outcomes. Our results suggest that the populations most affected by diseases are older, obese women with a history of heavy smoking, or who are still active heavy smokers.

The correlation between age and multimorbidity is well-known—due to the ageing of world population, the proportion of elderly people has increased steadily and, consequently, the global burden of diseases has increased. The older a patient is, the more likely they will develop another disease, resulting in a clustering of diseases in elderly patients [17]. Most of the comorbidities assessed in this study showed an age distribution that fits the described trend, but there were some exceptions. Perennial and seasonal rhinitis showed an inverse trend, which does align with the known age distribution of allergic diseases [18]. The proportion of patients diagnosed with perennial rhinitis was more than two-thirds of all our patients, which is much higher compared with the general population, and even to subsets of asthmatic populations examined in earlier studies [18, 19]. One of the reasons behind this finding could be that unlike all other comorbidities, the reporting of perennial rhinitis was based on symptoms and self-reporting, and not on exact detection of allergies. This approach could lead to an overestimation of the disease prevalence.

The higher proportion of female patients and the different prevalence of certain comorbidities are both well-documented aspects of asthmatic populations [4, 20–23]. Veenendaal et al. [20] examined the prevalence of comorbidities of more than 32,000 asthmatic patients and showed that females are affected by overall comorbidities at a higher rate compared with males, with significant differences in certain disease categories. Other studies have also presented a significant gender-related difference in certain comorbidities, including hypertension, cardiovascular diseases, depression, osteoporosis, and allergic diseases [21–23]. Most of our findings correlate with these data; however, in our study the prevalence of perennial rhinitis did not differ between the genders. The prevalence of GERD was markedly higher in females, contrary to the general population, where there is no difference between the genders [24]. On the other hand, COPD was more prevalent in males, an outcome that might be due to the higher prevalence of male active smokers in Hungary [25, 26]. AMI and atrial fibrillation were the only cardiovascular diseases that affected a higher percentage of male patients, but these diseases had the lowest prevalence in our population (1.65% and 1.26%, respectively). These low values could have affected the accuracy of the calculations.

Obesity is an important and thoroughly studied risk factor for asthma and a plethora of other diseases, such as hypertension, DM, and IHD among others [10, 22]. A very high proportion of our patient population was overweight (70%), even compared with the general Hungarian population, which is in the top 10 most obese countries in Europe [27, 28]. Due to the cross-sectional design of our study, it was not possible to determine whether obesity or asthma developed earlier in our patients. However, as our results show, obesity by itself worsens asthma control, and it is a well-known risk factor of other diseases, which makes asthma treatment even more difficult. It is paramount for patients to understand that maintaining a healthy weight is an important aspect of asthma control.

The influence of time since the asthma diagnosis is harder to interpret. On the one hand, when it had been a long time since the diagnosis, chronic disease clearly increases the risk of developing other diseases. On the other hand, there is a notable difference in the effect of severe and mild asthma on asthma control, which is independent of the time since diagnosis. Moreover, asthma is generally considered an underdiagnosed disease, a factor that could lead to more patients having a recent diagnosis (meaning a shorter time since diagnosis) despite the fact that they had underlying asthma for a longer time [29]. Untreated, the complications of asthma could be more severe and prevalent. Considering all these effects, our mixed results are easier to understand.

Smoking is not just an important risk factor for developing asthma; it is also one of the leading causes of preventable deaths in Hungary [30]. As a parallel to increasing the chance of having asthma, active smoking is also an important contributor to uncontrolled asthma [31]. We observed this dose-dependency in our findings, with a higher proportion of uncontrolled patients in the active, heavy-smoker groups. Perhaps the most interesting findings of our study were the results of the analysis of the effect of smoking on comorbid conditions. The prevalence of some diseases (like COPD) showed a clear, dose- and exposure-dependent correlation, which meant that the more a patient smoked, the more likely they were to have COPD; this increased risk was mitigated by smoking cessation. On the other hand, the effect of smoking status and exposure on the prevalence of some conditions showed a different pattern: in some cases different smoking habits (for example, starting age, time of cessation, intensity of smoking, and overall exposure) lead to different coexisting conditions. For example, both IFG and DM had a higher prevalence in heavy, ex-smokers compared with all other groups. These data could mean that different smoking habits could lead to different comorbidities, however we have to highlight that the differences could be caused by confounding variables not assessed in this study. Nonetheless these results suggest that a simple dose-dependent model is not suitable to describe accurately the relationships of all asthma comorbidities and smoking history, or at the very least it is necessary to weight the exposure based on the age of smoking initiation. Previous studies about the extent of the increased risk of developing certain diseases in people who start smoking at a younger age were inconclusive. However, Li et al. reported a marked increase in all-cause mortality in young smokers compared with adults [32, 33]. Despite this finding, there is a need for further, more detailed analysis.

Comorbidities can affect asthma control for two distinct reasons. First, some comorbidities have similar symptoms as asthma. Among others, COPD and IHD could cause coughing, shortness of breath, and decreased physical fitness, all of which could be attributed to asthma and, as such, could result in a patient being classified as ‘uncontrolled’ [1, 34]. This change of status may lead to escalation of therapy, which would not decrease the symptoms, because these are not caused by asthma. Keeping this chain of undesirable outcomes in mind, even the 2014 GINA guidelines recommended that physicians should check the treatment of comorbid conditions before treatment escalation [6]—an important recommendation still present in the GINA 2021 guideline [1]. The results of our study underline the importance of this recommendation because the aforementioned comorbidities were amongst the ones with the strongest effect on asthma control levels.

Another way to influence disease control is through the modification or enhancement of the disease pathogenesis, or the decrease of the response to otherwise adequate asthma treatment. Smoking and, consequently, COPD worsens asthma through decrease of lung function and increase of exacerbation risk and modification of the inflammatory reaction. GERD can also increase exacerbation risk and promote bronchoconstriction [11]. Allergic airway diseases, obstructive sleep apnoea, and psychopathologic conditions have also been reported to worsen asthma pathophysiology and decrease responsiveness to treatment [10, 11]. The findings in our study correlated with these data but due to the study design, it was not possible to distinguish the reasons behind worsening asthma control based on the aforementioned effects.

The most important strength of our study is the large sample size and the diversity of patients enrolled from all regions of Hungary: out of all registered adult asthmatics, 4.3% were enrolled by pulmonologists of 187 treatment centres. Due to the high number of treatment centres, our population can be considered representative of the Hungarian asthmatic population. Another strength of our study is the large number of different comorbidities assessed through a wide spectrum of disease categories.

The limitations of our study mainly originate from its cross-sectional nature, which did not allow the follow-up of patients and made it impossible to assess time-dependent relationships between parameters (for example, the identification of the sequence of development of comorbid conditions). An important limitation was the lack of further evaluation of the ‘other comorbidities’ disease group. Some important and notable comorbidities were not individually assessed (such as depression, anxiety, obstructive sleep apnoea, and bronchiectasis—all of which are recognised comorbidities of asthma). Assessment of those comorbidities would have been more impactful, and could have provided new insights into understudied, but important comorbid conditions.

## Conclusions

Our study has shown in a large, real-life patient cohort that most asthmatics have at least one comorbidity, the exact type of which is highly dependent on the patient’s basic characteristics, such as age or gender. Moreover, the presence of the most frequent comorbidities has a considerable impact on asthma control levels. Thus, the appropriate treatment of comorbidities is crucial before escalating asthma treatment.

## Data Availability

The datasets used and/or analysed during the current study are available from the corresponding author on reasonable request.

## Abbreviations

AF: Atrial fibrillation
BMI: Body mass index
CI: Confidence intervals
COPD: Chronic obstructive pulmonary disease
CV: Cardiovascular
DM: Diabetes mellitus
GINA: Global Initiative for Asthma
GCP: Good clinical practice
GERD: Gastroesophageal reflux disease
HT: Hypertension
IGT: Impaired glucose tolerance
ICS: Inhaled corticosteroid
IHD: Ischaemic heart disease
OR: Odds ratio
SABA: Short-acting beta-agonist
